# Topiramate is more effective than acetazolamide at lowering intracranial pressure

**DOI:** 10.1177/0333102418776455

**Published:** 2018-06-13

**Authors:** William J Scotton, Hannah F Botfield, Connar SJ Westgate, James L Mitchell, Andreas Yiangou, Maria S Uldall, Rigmor H Jensen, Alex J Sinclair

**Affiliations:** 1 Metabolic Neurology, Metabolic Neurology, Institute of Metabolism and Systems Research, University of Birmingham, Edgbaston, Birmingham, UK; 2 Centre for Endocrinology, Diabetes and Metabolism, Birmingham Health Partners, Birmingham, UK; 3 Department of Neurology, University Hospitals Birmingham NHS Foundation Trust, Birmingham, UK; 4 Institute of Inflammation and Ageing, University of Birmingham, Edgbaston, Birmingham, UK; 5 Danish Headache Center, Clinic of Neurology, Rigshospitalet-Glostrup, University of Copenhagen, Glostrup, Denmark

**Keywords:** Cerebrospinal fluid, idiopathic intracranial hypertension, headache, choroid plexus

## Abstract

**Background:**

The management of idiopathic intracranial hypertension focuses on reducing intracranial pressure to preserve vision and reduce headaches. There is sparse evidence to support the use of some of the drugs commonly used to manage idiopathic intracranial hypertension, therefore we propose to evaluate the efficacy of these drugs at lowering intracranial pressure in healthy rats.

**Methods:**

We measured intracranial pressure in female rats before and after subcutaneous administration of acetazolamide, topiramate, furosemide, amiloride and octreotide at clinical doses (equivalent to a single human dose) and high doses (equivalent to a human daily dose). In addition, we measured intracranial pressure after oral administration of acetazolamide and topiramate.

**Results:**

At clinical and high doses, subcutaneous administration of topiramate lowered intracranial pressure by 32% (*p* = 0.0009) and 21% (*p* = 0.015) respectively. There was no significant reduction in intracranial pressure noted with acetazolamide, furosemide, amiloride or octreotide at any dose. Oral administration of topiramate significantly lowered intracranial pressure by 22% (*p* = 0.018), compared to 5% reduction with acetazolamide (*p* = >0.999).

**Conclusion:**

Our *in vivo* studies demonstrated that both subcutaneous and oral administration of topiramate significantly lowers intracranial pressure. Other drugs tested, including acetazolamide, did not significantly reduce intracranial pressure. Future clinical trials evaluating the efficacy and side effects of topiramate in idiopathic intracranial hypertension patients would be of interest.

## Introduction

Idiopathic intracranial hypertension (IIH) typically affects obese woman of childbearing age and is characterised by raised intracranial pressure (ICP). Morbidity results from chronic disabling headaches and papilloedema, with the potential for severe visual loss (permanent in up to 25%) (1). IIH affects 1–2 per 100,000 of the general population and 20 per 100,000 of the obese female population with numbers expected to rise over the forthcoming decade in line with escalating obesity figures (2). Management strategies focus on disease modification through weight loss, although this is notoriously difficult to achieve (3). Therefore, the majority of patients receive pharmacological therapy with the aim of reducing cerebrospinal fluid (CSF) secretion and consequently ICP. For those with fulminant IIH and rapidly declining vision, CSF diversion surgery may be necessary (4).

Acetazolamide is the most commonly used drug in IIH. Class 1 evidence has demonstrated modest improvement in visual ﬁeld function in patients with IIH with mild visual loss (5,6). However, the 2015 Cochrane review (7) has summarised that there is currently insufficient evidence to recommend or reject the efficacy of acetazolamide for treating IIH. Between 19–48% of patients will not tolerate acetazolamide due to side effects (5,6) and consequently alternative drugs maybe prescribed such as topiramate, furosemide, amiloride and octreotide. However, there is extremely limited mechanistic and clinical data to support their use. The purpose of this study was to determine which drug currently used to treat IIH has the greatest effect at lowering ICP in rats to provide pre-clinical evidence for its use in IIH.

## Materials and methods

### Experimental animals

Female Sprague-Dawley (SD) rats (Taconic, Denmark) weighing 150–250 g were used in this study. The rats were maintained in cages kept under a 12-hour light/dark cycle with free access to food and water. All experimental procedures were approved by the Danish Animal Experiments Inspectorate (license number 2014-15-0201-00256) and comply with the ARRIVE guidelines. After treatments and surgical procedures, the rats were monitored daily for any adverse effects.

### Drugs

The dose of the drug was determined using the 2005 FDA guidance for industry, which describes how to estimate the maximum safe starting dose in healthy volunteers (8): Human equivalent dose (mg/kg) = rat drug concentration (mg/kg)/6.2. Therefore, to convert the human dose to an equivalent rat dose we used the equation: rat drug concentration (mg/kg) = 6.2 × human dose (mg/kg based on a 60 kg human). The rat clinical dose was calculated using the human single dose, and the rat high dose was equivalent to the human daily dose (Table 1).

**Table 1.** Human and rat equivalent doses*.

Drug	Human single (clinical) dose	Human daily (high) dose	Rat clinical dose	Rat high dose	Rat Tmax (T½) hours	Reference
Subcutaneous						
Topiramate	50 mg	200 mg	5.2 mg/kg	20.6 mg/kg	0.7 ± 0.5 (2.5) 20 mg/kg oral	(11)
Acetazolamide	1 g	4 g	103.3 mg/kg	413.4 mg/kg	1–3 (6) oral	(12)
Amiloride	5 mg	20 mg	516.7 µg/kg	2.0 mg/kg	4 (21.7)** 10 mg/kg oral	(13)
Octreotide	350 µg	2 mg	36.2 µg/kg	206.6 µg/kg	1 (0.7 ± 0.1) 500 µg/kg SC	(14)
Furosemide	40 mg	240 mg	4.1 mg/kg	24.8 mg/kg	1 (0.5) 40 mg/kg oral	(15)
Oral						
Topiramate	–	200 mg	–	6.25 mg/rat		
Acetazolamide	–	4 g	–	125 mg/rat		

* Rat drug concentration (mg/kg) = 6.2 × human dose (mg/kg based on a 60 kg human).

** Tmax for subcutaneous amiloride is not known but is expected to be less than the oral Tmax.

Previous studies investigating the effects of drugs on ICP administered them via various routes; however, in this study we standardised the delivery route to subcutaneous injection before going on to assess the most promising drugs via their usual route of administration (oral). Acetazolamide (A6011, Sigma-Aldrich), furosemide (F4381, Sigma-Aldrich) and topiramate (13623, Cayman Chemical) were initially dissolved in NaOH and then the pH lowered to 8.7, 7.7 and 7.8 respectively, with hydrochloric acid (HCl). Amiloride HCl (129876-100, Merck Millipore) and Octreotide acetate salt (H-5972, Bachem) were dissolved in sterile water. The stock solutions were further diluted in 0.9% sodium chloride (NaCl) to their final concentrations for subcutaneous injection. Hyperosmolar solutions are known to have ICP lowering effects (9), therefore we measured the sodium and chloride concentrations of each drug in solution. The osmolarity of the clinical dose acetazolamide solution was equivalent to 2% NaCl, and the high dose acetazolamide solution was equivalent to 4% NaCl. The topiramate, amiloride, octreotide and furosemide solutions’ osmolarity were equivalent to 0.9% NaCl. For this reason, we compared the clinical and high dose acetazolamide against controls of 2% and 4% NaCl respectively. All other drugs were compared against a 0.9% NaCl control. Acetazolamide tablets (250 mg) and topiramate tablets (25 mg) were purchased from the Danish pharmacy supply and split into half and a quarter respectively for oral dosing. The drug was ground into a powder and mixed with Nutella®, and Nutella® by itself was used as the placebo.

### Epidural ICP probe implantation

Implantation of an epidural ICP probe is described in full elsewhere (10). Briefly, the rats were anaesthetised (2.7 ml/kg subcutaneous injection containing 1.25 mg/ml midazolam, 2.5 mg/ml fluanisone and 0.079 mg/ml fentanylcitrate) and placed in a stereotactic frame (David Kopf Instruments) and the bone was exposed by retracting the skin and soft tissue. A large burr hole was carefully drilled to expose the dura mater to enable placement of the epidural ICP probe (PlasticsOne). Three other small burr holes were made to fit anchoring screws to the skull and the epidural ICP probe was secured using dental resin-cement (Clearfil SA Cement, RH Dental). The epidural ICP probe and the transducer (DTX-Plus™, Argon Medical Devices) were then connected by a polyethylene tube filled with sterile water. The pressure signal was visualised and recorded using Perisoft v.2.5.5 (Perimed). Correct ICP signal was confirmed by the transient elevation of ICP after jugular vein compression. When the ICP recording procedure was completed, the epidural ICP probe was closed with a bite proof cap (PlasticsOne) and the rat allowed to recover.

### Drug administration

Subcutaneous drug administration: On day 0, the epidural ICP probe was implanted and the rat allowed to recover. On days 3, 6, 9, 12, 15, 18 and 21, ICP recordings with drug treatments were conducted while the rats were sedated with midazolam (2.5 mg/kg subcutaneous injection) in an infusion cage (Instech Laboratories), which had a swirl lever arm to ensure unhindered movement. A stable baseline ICP reading was recorded for around 30 minutes, before the rats received a subcutaneous injection of the drug. ICP was recorded for a further 120 minutes, after which the rat was returned to its normal cage (this includes the peak plasma concentration (Tmax) for the majority of the drugs; Table 1 (11–15)). A randomised cross over block design was used to allocate the order of drug treatment and the dose. Towards the end of the experiment it was increasingly difficult to measure ICP in some of the rats, possibly due to blockage or scarring of the epidural ICP probe, and these measurements were excluded from analysis. The final numbers for each group are included in the figure legend (a minimum of n = 5 in each treatment group and n = 4 in control groups).

Oral drug administration: On day 0, the epidural ICP probe was implanted and the animal allowed to recover followed by 12-hour ICP recordings (to include the Tmax) on day 3 (conducted as above). Rats were randomly allocated to receive placebo, 125 mg acetazolamide tablet or 6.25 mg topiramate (high doses), crushed in Nutella®. This resulted in n = 5 in each treatment group. Water intake was measured over the course of the experiment, and at 12 hours the animals were sacrificed, with blood and CSF samples taken for pH measurement.

### Statistical analysis

Sample numbers were calculated using a power calculation based on our previous results with acetazolamide (16). The data was assessed for normality and the values were represented as mean and standard error of the mean (SEM). The data was analysed using GraphPad Prism software (v.7). For comparison of ICP area under the curve (AUC) for NaCl 0.9% versus clinical and high drug doses (topiramate, furosemide, amiloride and octreotide), one-way ANOVA (followed by post-hoc Tukey test to correct for multiple comparisons) was used. For comparison of ICP AUC for NaCl 2% versus acetazolamide clinical dose, and NaCl 4% versus acetazolamide high dose, unpaired T-tests were used. Values were considered statistically significant when *p* values were less than 0.05.

## Results

### Subcutaneous dosing and effect on ICP

**Figure 1.**
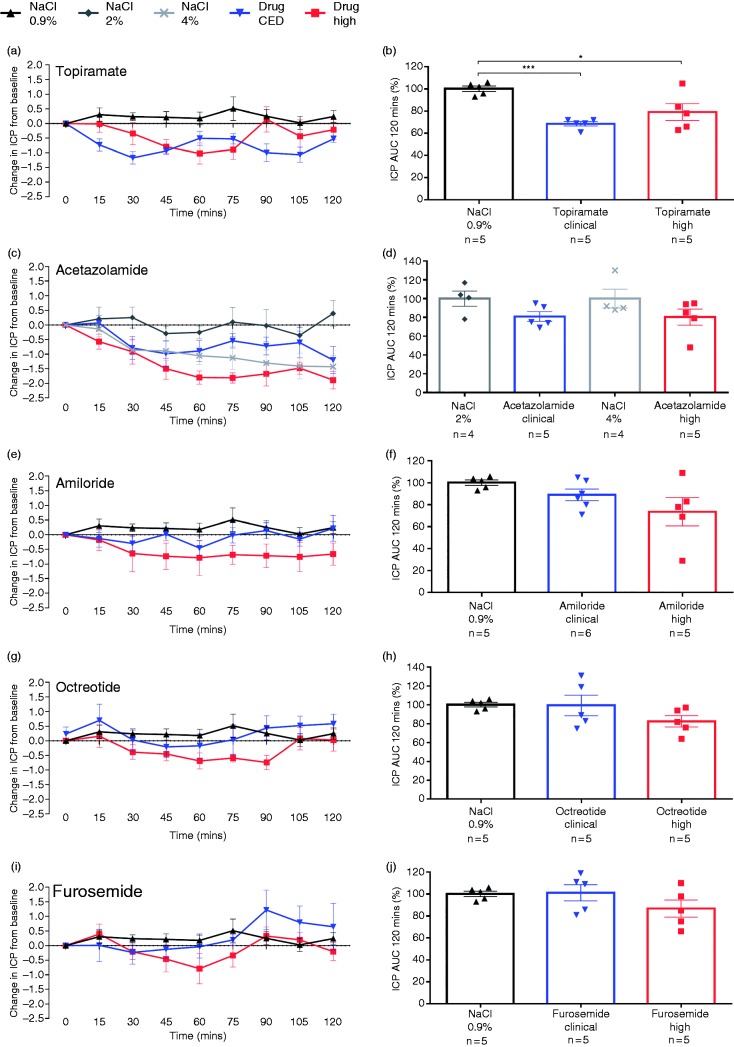
Effect of subcutaneous administration of clinical or high dose drugs on ICP. Rats received a subcutaneous injection of topiramate ((a) and (b)), acetazolamide ((c) and (d)), amiloride ((e) and (f)), octreotide ((g) and (h)) and furosemide ((i) and (j)). ((a), (c), (e), (g), (i)) Line graphs showing the change in ICP from baseline (mmHg) ± SEM, after subcutaneous injection with either high or clinical dose of drug measured every 15 minutes for 120 minutes. ((b), (d), (f), (h), (j)) Bar graphs showing percentage of control ICP AUC over 120 minutes ± SEM, with clinical or high dose of drug. Controls: 2% NaCl for acetazolamide clinical dose, 4% Na Cl for acetazolamide high dose, and 0.9% NaCl for all other drugs tested.

At clinical and high doses, subcutaneous administration of topiramate significantly lowered ICP over 2 hours to 68.6 ± 2.0% of baseline (32% reduction, *p* < 0.001) and 79.2 ± 7.5% of baseline (21% reduction, *p* < 0.05) respectively compared to 0.9% NaCl control (Figure 1(a) and (b), and Table 2). Subcutaneous administration of acetazolamide showed a trend towards a reduction in ICP at clinical (19% reduction) and high doses (20% reduction); however, it was not significantly different compared to the 2% and 4% NaCl controls respectively (Figure 1(c) and (d), and Table 2). At both clinical and high doses the other drugs, amiloride (10% and 27% reduction respectively), octreotide (1% and 18% reduction respectively), and furosemide (1% and 13% reduction respectively), did not significantly reduce ICP compared to 0.9% NaCl control (Figure 1(e)-(j), and Table 2).

**Table 2.** Summary of results.

Drug	Clinical dose		High dose	
% change	*p* value	% change	*p* value
Subcutaneous				
Topiramate	−32%	0.0009 (***)	−21%	0.015 (*)
Acetazolamide	−19%	0.08 (ns)	−20%	0.18 (ns)
Amiloride	−10%	0.51 (ns)	−27%	0.08 (ns)
Octreotide	−1%	>0.99 (ns)	−18%	0.19 (ns)
Furosemide	−1%	0.99 (ns)	−13%	0.28 (ns)
Oral				
Topiramate	–	–	−22%	0.018 (*)
Acetazolamide	–	–	−5%	>0.999 (ns)

All results analysed with 1-way ANOVA, except 2% NaCl vs. acetazolamide clinical dose, and 4% NaCl vs. acetazolamide high dose, which were analysed with unpaired T-tests. All statistical analysis and graphs performed in Graph Prism. Values were considered statistically significant when *p* values were *p < 0.05, ***p < 0.001.

### Oral dosing and effect on ICP

**Figure 2.**
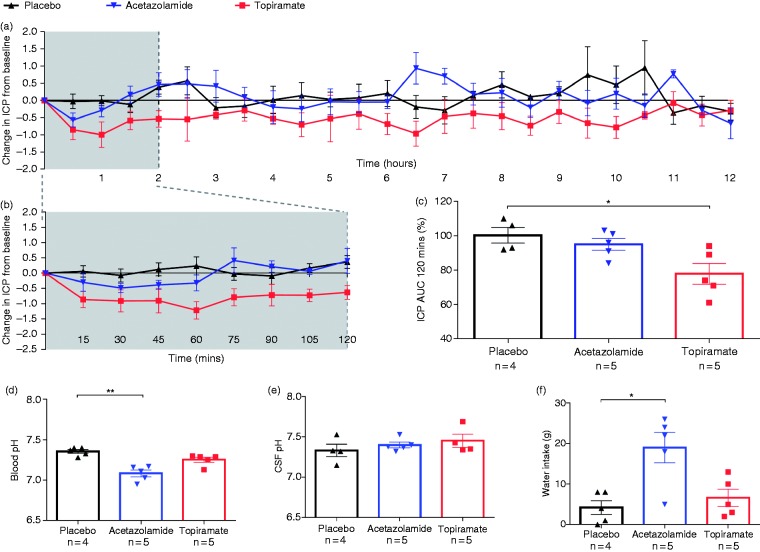
Effect of oral dosing of acetazolamide or topiramate on ICP, CSF pH, blood pH and water intake. (a) Line graph showing the change in ICP from baseline (mmHg) ± SEM, measured every 30 minutes for 12 hours after treatment; (b) line graph showing the change in ICP from baseline (mmHg) ± SEM, measured every 15 minutes for 120 minutes after treatment; (c) bar graph showing percentage of control ICP AUC over 120 minutes ± SEM after treatment. Bar graphs showing blood pH (d), CSF pH (e) and water intake, (f) at 12 hours after treatment.

Oral administration of topiramate lowered ICP, reaching a maximum after 1 hour (1.0 ± 0.4 mmHg reduction in ICP) and returning to baseline at 11 hours (0.1 ± 0.3 mmHg reduction in ICP) (Figure 2(a)). Over the first two hours, topiramate significantly reduced ICP to 77.8 ± 6.0% of baseline AUC (22% reduction, *p* < 0.05), compared to placebo. Acetazolamide did not significantly lower ICP over this period (95.0 ± 3.5% of baseline AUC, 5% reduction) (Figure 2(a)–(c), and Table 2).

### Oral dosing and effect on blood pH, water intake and CSF pH

Acetazolamide significantly lowered blood pH (7.08 ± 0.04 pH, *p* < 0.01), and led to increased water intake (19.0 ± 3.7 g, *p* < 0.05), but had no effect on CSF pH (7.40 ± 0.04 pH) compared to placebo (7.36 ± 0.02 pH, 4.2 ± 1.7 g, 7.33 ± 0.08 pH respectively). Topiramate had no significant effect on blood pH (7.25 ± 0.03 pH), water intake (6.6 ± 2.1 g) or CSF pH (7.45 ± 0.08 pH) (Figure 2(d)–(f)).

## Discussion

The aim of the present study was to ascertain which drug – acetazolamide, topiramate, furosemide, amiloride or octreotide – had the greatest effect on lowering ICP in healthy rats, and so provide pre-clinical evidence to support their use in the pharmacological treatment of IIH. We were able to demonstrate that both subcutaneous and oral administration of topiramate significantly reduced ICP. In addition, our results suggest that acetazolamide, the current first line oral therapy in IIH, does not significantly lower ICP. Amiloride, octreotide, and furosemide also had no effect on ICP when administered subcutaneously.

**Figure 3.**
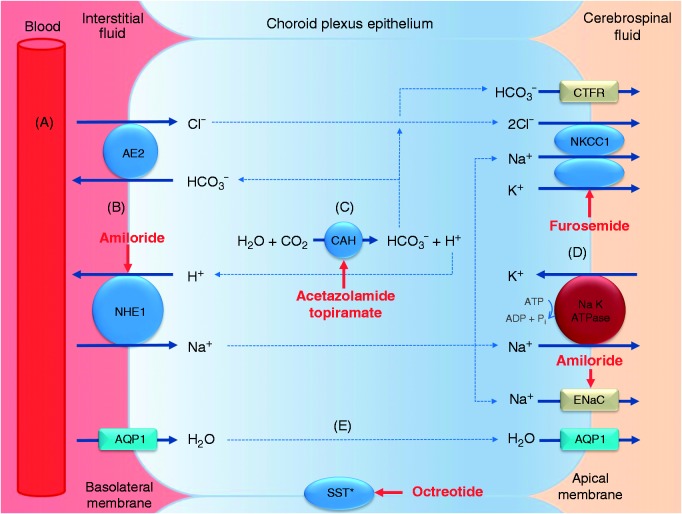
CSF formation in the choroid plexus and potential sites of action of the commonly used drugs in IIH. (a) Hydrostatic pressure drives the passive filtration of fluid from the blood through the fenestrated capillaries into the choroidal interstitial fluid. (b) At the basolateral membrane, ion exchangers substitute H^+^ and HCO_3_^−^ for Na^+^ and Cl^−^ respectively. (c) The carbonic anhydrase enzyme catalyses the conversion of H_2_O and CO_2_ to HCO_3_^−^ and H^+^. (d) On the apical surface, the Na^+^ K^+^ ATPase actively pumps 2K^+^ in and 3Na^+^ out and the Na^+^−K^+^−2Cl^−^ co-transporter, driven by the accumulation of Cl^−^, moves 2Cl^−^, Na^+^ and K^+^ ions out. HCO_3_^−^ and K^+^ also passively move out of the cells. (e) The net movement of Na^+^, Cl^−^ and HCO_3_^−^ generates an osmotic gradient causing the movement of water in the same direction. Water moves mainly via a transcellular route, with aquaporin 1 at the basolateral and apical membrane facilitating water transport along this osmotic gradient. CAH: carbonic anhydrase; SST: somatostatin receptor (*location in the choroid plexus unknown); AQP1: aquaporin 1; NKKC1: Na-K-CL cotransporter 1; NHE-1: Na-H antiporter 1; AE2: anion exchange protein 2; ENaC: epithelial Na channel.

The management of IIH is focused on reducing ICP to try to both preserve visual function, as well as reduce long-term headache disability. Pharmacological management is one option to reduce ICP, and current medications used are thought to principally work by reducing CSF secretion. CSF secretion involves numerous enzymes, ion channels and transporters that produce a net movement of ions across the choroid plexus epithelium, thus creating an osmotic gradient that drives water from the blood stream into the ventricles (17). The proposed mechanisms of action of the different drugs investigated in this study involve inhibition of these enzymes or transporters at various stages in this process (Figure 3).

Acetazolamide is the most commonly used first line drug for the treatment of IIH. It is a carbonic anhydrase (CA) inhibitor, which reduces ion transport and consequently water across choroid plexus epithelial cells (17) and can decrease CSF secretion by 57–64% (16,18). However, the evidence that acetazolamide reduces ICP and is an effective treatment for IIH is inconclusive. Intravenous infusion (19) and intraperitoneal injection (16) of acetazolamide in rats causes a reduction in ICP; however, intraventricular administration has no effect on ICP (20). In addition, hydrocephalic infants treated with intravenous acetazolamide showed a transient increase in ICP, while those treated with oral acetazolamide demonstrated no change in ICP (21). Our studies suggest that when compared to an appropriate vehicle control (4% NaCl), acetazolamide does not significantly reduce ICP in our model. We suggest that part of the ICP lowering effect of acetazolamide seen in previous animal studies may be due to the solution it is dissolved in, as hypertonic solutions can lower ICP (9). Furthermore, in this study oral administration of acetazolamide, the most clinically relevant delivery route as the drug is given orally in IIH patients, lowered blood pH and induced diuresis but did not lower ICP. We do acknowledge, however, that the diuresis noted in the rodents on acetazolamide is the result of inhibition of renal carbonic anhydrase, reflecting systemic absorption, and cannot be extrapolated to infer the extent of inhibition of carbonic anhydrase at the choroid plexus. There are currently only two randomised control trials in IIH comparing acetazolamide to placebo (5,6). They demonstrate modest improvements in IIH symptoms; however, many patients are unable to tolerate high doses of acetazolamide due to a plethora of side effects including fatigue, paraesthesia, and gastrointestinal symptoms. As such, a Cochrane review (2015) concluded that there is insufficient evidence to recommend acetazolamide as a first line intervention for IIH (7).

We are the first to demonstrate that topiramate significantly reduces ICP after subcutaneous and oral administration in a rat model. Originally used as a treatment for epilepsy, it has many mechanisms of action within the CNS including being an effective CA inhibitor, with a similar inhibitory activity of CAII and CAXII isoforms to acetazolamide (inhibition constants CAII – 10 nM topiramate vs. 12 nM acetazolamide; CAXII – 3.8 nM topiramate vs. 5.7 nM acetazolamide) (22). In addition, it is more lipophilic than acetazolamide and therefore is likely to have higher intracellular concentrations. Topiramate also has additional properties that make it of particular interest in the treatment of IIH. Class 1 evidence shows that topiramate is an effective prophylactic treatment for both episodic and chronic migraine (23–25), and given the prevalence of headache in IIH patients, this prophylactic action may be beneficial. Another well-documented effect of topiramate is weight loss (22,26,27), which would be of particular interest in the treatment of IIH as weight loss has already been shown to significantly reduce ICP, headaches and papilloedema (3). Several case studies have demonstrated improvements in IIH symptoms with topiramate (28–30). However, the putative benefits of topiramate over acetazolamide would need to be weighed up in relation to its well-documented side effects, which include paraesthesia, cognitive impairment, fatigue, insomnia, anxiety and nephrolithiasis. However, the discontinuation rate of topiramate was only 20–30% in the treatment arm of a chronic migraine randomised clinical trial (31), which compares favourably with the discontinuation rates seen with acetazolamide in the IIH RCT by Ball et al. (5).

Furosemide inhibits the Na^+^-K^+^-2Cl^−^ cotransporter (NKCC1) on choroid plexus epithelial cells, and in animal studies reduces CSF secretion by 20–50% (32,33). Amiloride blocks the Na^+^/H^+^ exchanger and/or Na^+^ channels (ENaC) on choroid plexus epithelial cells and may also act upon blood vessels of the choroid plexus to alter CSF secretion. In animal models, amiloride has been shown to reduce CSF secretion by up to 50%, though only when administered into the carotid artery (34,35). Although previous studies demonstrate furosemide and amiloride alter CSF secretion, our study suggests neither drug significantly reduces ICP.

Octreotide, a somatostatin analogue, is predominantly used to manage growth hormone-releasing pituitary tumours. Recently, two uncontrolled studies conducted in IIH patients have shown an improvement in IIH symptoms with octreotide treatment (36,37). As somatostatin receptors are highly expressed in the arachnoid villi and choroid plexus, it was proposed that octreotide could directly influence CSF dynamics and thus ICP (38). Our studies do not support this hypothesis, instead showing that subcutaneous administration of octreotide does not alter ICP. However, the beneficial effects of octreotide observed in IIH patients could be due to its anti-obesity properties (39).

There are several limitations to our study that need to be addressed. Firstly, we only assessed the effect of each of the drugs on ICP after one administration and cannot exclude that the effects of the drugs on ICP may alter with repeated dosing. However, we have monitored the ICP over the duration of the peak plasma concentrations (Tmax) and consequently would predict an effect within this time frame, as is seen with other therapeutic agents that alter ICP (40). Secondly, these studies were conducted in rats and we do not know whether these results will directly translate to humans. However, both mannitol and hypertonic saline have been shown to reduce ICP in rats and humans (40,41). The high dose used for acetazolamide was based on the clinically relevant highest dose of 4 g that 40% of the patients were on in the Idiopathic Intracranial Hypertension Treatment Trial (IIHTT), which equated to roughly 80 mg per rat, significantly less than doses used in previous animal studies (16). It is possible that if higher doses were used (although these would be less clinically relevant), a greater effect could have been seen as noted in other papers. In addition, if a repeated dosing regimen was used, as in the IIHTT study, a higher steady state concentration of acetazolamide would likely have been reached, compared to our model where only a single dose was used. This is obviously also true of the other drugs, however, including topiramate. The final limitation is that we used healthy rats, rather than a disease model of raised ICP. There are reports that CSF secretion may change in raised ICP models (42); however, our previous studies investigating the GLP-1 R agonist exendin-4 demonstrate that drug-induced reductions in ICP seen in healthy rats are replicated in a model of hydrocephalus (43).

In summary, out of all the drugs we tested that are currently used to treat IIH, only topiramate significantly reduced ICP. Topiramate may have additional advantages in IIH, including its migraine prevention properties and weight loss effects; however, the side effects can be considerable. Future studies comparing the physiological effects of these drugs to reduce ICP in IIH patients would be of interest.

## Article highlights

- There is limited evidence to support the use of some of the drugs commonly used to manage idiopathic intracranial hypertension.
- We demonstrate that topiramate reduces intracranial pressure at clinically relevant doses.
- At clinically relevant doses, acetazolamide, furosemide, amiloride and octreotide do not lower intracranial pressure.
